# Expression and significance of PD-1 and PD-L1 in patients with recurrent spontaneous abortion: A protocol for systematic review and meta-analysis

**DOI:** 10.1097/MD.0000000000025444

**Published:** 2021-04-09

**Authors:** Tao Li, Yihong Chen, Yi Lai, Guoqian He, Guolin He

**Affiliations:** a Key Laboratory of Birth Defects and Related Diseases of Women and Children, Ministry of Education; b Department of Obstetrics and Gynecology; c Department of Pediatrics, West China Second University Hospital, Sichuan University, Chengdu, Sichuan, China.

**Keywords:** meta-analysis, programmed death-1, programmed death ligand-1, protocol, recurrent spontaneous abortion

## Abstract

**Background:**

Recurrent spontaneous abortion (RSA) accounts for the most common complication of early pregnancy in humans. As an immune checkpoint pathway, programmed death-1 (PD-1) and programmed death ligand-1 (PD-L1) can be exploited by tumor cells to evade immuno-surveillance. Many studies have shown that the expression of PD-1/PD-L1 is involved in RSA. However, the correlation between the expression of PD-1/PD-L1 and RSA is still controversial. We conducted meta-analysis to further explore the correlation between the expression of PD-1/PD-L1 and RSA, to provide a basis for clinical prevention and treatment.

**Methods:**

We will search PubMed, Embase, Web of Science, Google Scholar, Chinese National Knowledge Infrastructure, Chinese VIP Information, Wanfang Database, and Chinese Biomedical Literature Database for related published studies before February 2021. Two review authors will search and assess relevant studies independently. Case control studies and cohort studies will be included. The Revman 5.3 software was applied to carry out the meta-analysis for the included literature.

**Results:**

The findings of this systematic review will be disseminated in a peer-reviewed publication and/or presented at relevant conferences.

**Conclusion:**

This study will provide a new theoretical basis for the prevention and treatment of RSA.

**Trial registration number:**

DOI 10.17605/OSF.IO/CZD23.

Ethics and dissemination: Formal ethical approval is not required, as the data are not individualized.

## 1 Introduction

Recurrent spontaneous abortion (RSA) means that spontaneous abortion occurs more than 2 times in a row, accounting for 1% to 2% of the entire group of women of gestational age.^[1–3]^ The common causes of RSA are uterine abnormalities or uterine adhesions, luteal insufficiency or hypothyroidism, infection factors such as mycoplasma, chlamydia, and cytomegalovirus, as well as autoimmune disorders, malnutrition, environmental factors, and so on.^[4,5]^ However, there are still about 50% of patients with unknown cause, which is called recurrent miscarriage of unknown cause.^[6]^

In recent years, more and more attention has been paid to the role of immunological factors in RSA. Fetal genes are determined by both paternal and maternal lines, and pregnancy, as a semi-allogeneic transplantation process, generally needs to maintain immune balance through effective immune regulation to avoid abortion caused by maternal immune system rejection.^[7]^ Therefore, immune imbalance plays an important role in RSA. Among them, lymphocyte immunotherapy as a common clinical treatment, can improve the immune imbalance of patients, and the curative effect is recognized.^[8]^

Programmed death-1 (PD-1) and programmed death ligand-1 (PD-L1) are the current focus of immunotherapy and they play an important role in T lymphocyte-mediated cellular immunity.^[9–11]^ In addition, some studies have shown that PD-1/PD-L1 was abnormally expressed in renal cell carcinoma, lung cancer, bladder cancer and other malignant tumors, and may be involved in the induction of maternal immune tolerance and the maintenance of pregnancy.^[12]^

PD-1 and PD-L1 mainly play an important role in T cell immune response and immune homeostasis.^[13,14]^ After they are combined, they play a negative regulatory role in the immune response of the body through the immunoreceptor tyrosine inhibitory motifs to introduce inhibitory signals, inhibit the proliferation and activation of T cells, and inhibit the survival of T cells. In addition, it can promote the production and differentiation of regulatory T cells and enhance the function of regulatory T cells. T lymphocytes, as one of the important immune cells, affect the formation of maternal-fetal tolerance during pregnancy, while abnormal activation of effector T cells and decreased inhibition of regulatory T cells will lead to RSA.^[15,16]^

The abnormal expression of PD-1/PD-L1 plays an important role in the occurrence and development of RSA, which clearly indicates that the expression of PD-1/PD-L1 can be applied as a biomarker to evaluate the risk of RSA. Many studies have explored the relationship between the expression of PD-1/PD-L1 and the risk of RSA.^[17–21]^ However, the results of these studies are not consistent. Therefore, we conducted a meta-analysis to examine the accurate correlation between the expression of PD-1/PD-L1 and susceptibility to RSA.

### 1.1 Protocol register

This protocol of systematic review and meta-analysis has been drafted under the guidance of the preferred reporting items for systematic reviews and meta-analysis protocols.^[22]^ Moreover, it has been registered on the OSF (registration number: DOI 10.17605/OSF.IO/CZD23).

### 1.2 Ethics

Since this is a protocol without patient recruitment and personal information collection, approval by the ethics committee is not required.

### 1.3 Eligibility criteria

Articles were included if they met the following criteria:

(1) Case-control, or cohort studies.
(2) All patients met the diagnostic criteria of RSA established by the American Society of Reproductive Medicine after medical history inquiry and systematic examination.^[23]^
(3) The control group included healthy women with no adverse pregnancy history.
(4) Results contained the evaluation of PD-1/PD-L1 expression.
(5) Language would be restricted to Chinese and English.

### 1.4 Exclusion criteria

(1) Animal-model studies, case reports, review articles, letters, comments, and editorials.
(2) The published papers were abstracts or the data were incomplete, and the papers with complete data were not available after contacting the author.

### 1.5 Information sources and search strategy

The search will use a sensitive subject and topic-based strategy from inception to February 2021. The searched database includes PubMed, Embase, Web of Science, Google Scholar, Chinese National Knowledge Infrastructure, Chinese VIP Information, Wanfang Database, and Chinese Biomedical Literature Database. Taking PubMed as an example, the retrieval strategy is demonstrated in Table 1.

**Table 1:** Search strategy in PubMed database.

Number	Search terms
#1	Abortion, Habitual[MeSH]
#2	Abortion, Recurrent[Title/Abstract]
#3	Miscarriage, Recurrent[Title/Abstract]
#4	Abortions, Habitual[Title/Abstract]
#5	Abortions, Recurrent[Title/Abstract]
#6	Habitual Abortion[Title/Abstract]
#7	Habitual Abortions[Title/Abstract]
#8	Miscarriages, Recurrent[Title/Abstract]
#9	Recurrent Abortion[Title/Abstract]
#10	Recurrent Abortions[Title/Abstract]
#11	Recurrent Miscarriage[Title/Abstract]
#12	Recurrent Miscarriages[Title/Abstract]
#13	Recurrent spontaneous abortion[Title/Abstract]
#14	or/1–13
#15	Programmed death-1[Title/Abstract]
#16	PD-1[Title/Abstract]
#17	Programmed death ligand-1[Title/Abstract]
#18	PD-L1[Title/Abstract]
#19	or/15–19
#20	#14 and #19

### 1.6 Data filtering and extraction

**Figure 1:**
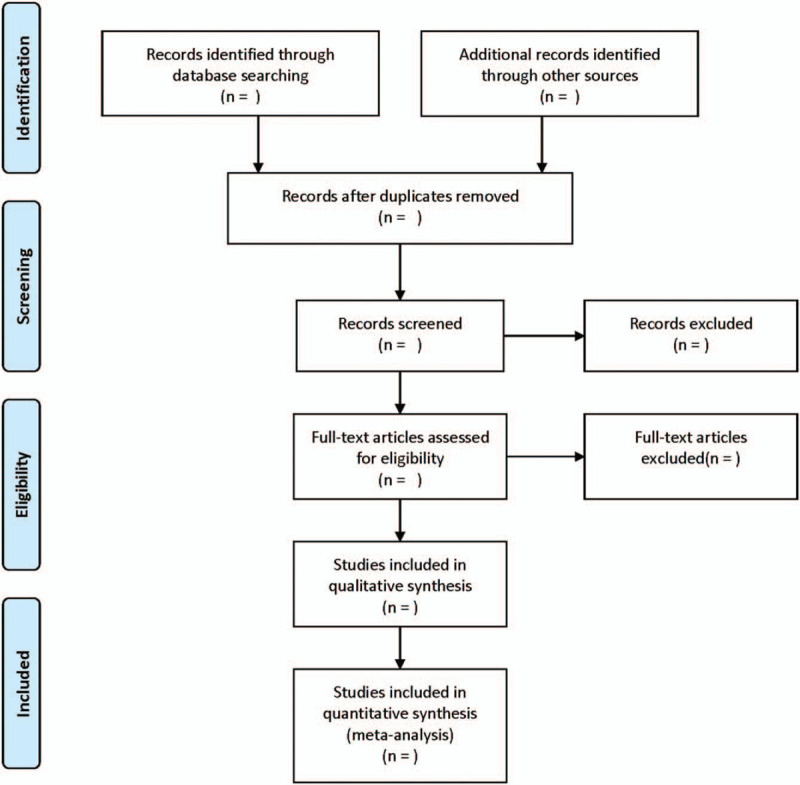
The process of literature filtering.

Two researchers independently complete the literature screening, exclude the studies that obviously do not meet the inclusion criteria, and further read the abstracts and the full texts to determine whether they meet the inclusion criteria. The data included in the literature will be extracted and cross-checked. Disagreement should be solved by consulting a third researcher, thus reaching a consensus. The extracted data include: first author, year, region, ethnicity, sample source, sample size, mean value, standard deviation value, assay, etc. The process of literature filtering is exhibited in Figure 1.

### 1.7 Literature quality assessment

As the studies involved in this meta-analysis are all case-control ones, the quality of the included studies was assessed by Newcastle–Ottawa scale.^[24]^ In this scale, “patient selection,” “comparability of study groups,” and “exposure” consist of a particular “star system” to evaluate the included studies. The lowest score was 0 star, and the highest score was 9 stars. Studies, with the score ≥5 stars, were defined as high quality. On the other hand, studies, with a score <5 stars, were defined as low quality. The quality of all included studies was assessed by 2 authors. Discrepancy was resolved through the discussion between the 2 authors.

### 1.8 Statistical analysis

#### 1.8.1 Data analysis and processing

A meta-analysis was conducted using RevMan 5.3. In the Cohen statistics of our association test, *P* (*P*-value of association), standard mean difference, and 95% confidence intervals were calculated. Heterogeneity was ascertained using *I*^2^. *I*^2^ < 50% revealed that the studies exhibited homogeneity, so fixed effects model was used. Otherwise, the random effects model was adopted. In the presence of heterogeneity, sensitivity analyses, and subgroup analysis would be conducted to investigate heterogeneity sources.

#### 1.8.2 Dealing with missing data

If there are missing data in the article, the author would be contacted via email for additional information. If the author cannot be contacted, or the author has lost relevant data, descriptive analysis will be conducted instead of meta-analysis.

#### 1.8.3 Sensitivity analysis

In order to test the stability of meta-analysis results of indicators, a one-by-one elimination method will be adopted for sensitivity analysis.

#### 1.8.4 Assessment of reporting biases

Publication bias was assessed by funnel plot that was performed for no less than 10 studies.^[25,26]^

#### 1.8.5 Subgroup analysis

We performed the subgroup analyses by factors of ethnicity and number of abortions.

## 2 Discussion

In order to maintain normal maternal pregnancy, blocking antibodies are mainly produced by embryonic human class II antigens and trophoblast cross antigens, and combine with maternal lymphoid antigens or placental trophoblast antigens, so as to prevent them from being recognized and killed by the maternal immune system, avoid the occurrence of harmful immune response, and finally achieve the purpose of maintaining normal pregnancy.^[27–30]^ Abnormal increase of PD-1/PD-L1 is not conducive to pregnancy, resulting in abortion.^[21]^

At present, many studies have revealed that a close relationship between PD-1/PD-L1 and the occurrence and development of RSA can be found, while the relationship between the expression of PD-1/PD-L1 and the occurrence of RSA is controversial.^[17–21]^ This study comprehensively searched the study on the relationship between the expression of PD-1/PD-L1 and the risk of RSA and made a quantitative comprehensive evaluation by carrying out meta-analysis to provide evidence-based reference for the study on the etiology of RSA.

This study also has some limitations. First of all, ethnic groups of meta-analysis is wide, while researches on single race are few. Population confounding factors may affect the credibility of the results in this study. Second, the method adopted in this study is meta-analysis that belongs to the secondary analysis. Therefore, the quality of this study mainly depends on the overall quality of the included studies, which is an inevitable problem of the secondary analysis. Third, due to the limitation of the number of studies included, there may be some publication bias in this meta-analysis.

To sum up, this meat-analysis will provide the correlation between the expression of PD-1/PD-L1 and the risk of RSA. In view of the limitation in terms of the number of included studies, this conclusion needs to be verified through further large sample and high-quality studies, so as to clarify the relationship between the expression of PD-1/PD-L1 and the risk of RSA.

## Author contributions

**Conceptualization:** Tao Li, Guolin He.

**Data curation:** Tao Li, Yihong Chen.

**Funding acquisition:** Guolin He.

**Investigation:** Guoqian He, Guolin He.

**Methodology:** Yihong Chen, Guoqian He, Guolin He.

**Project administration:** Guolin He.

**Resources:** Yihong Chen.

**Software:** Tao Li.

**Validation:** Yi Lai.

**Visualization:** Yi Lai.

**Writing – original draft:** Tao Li, Guolin He.

**Writing – review & editing:** Tao Li, Guolin He.
